# Plasmonic Enhancement in BiVO_4_ Photonic Crystals for Efficient Water Splitting

**DOI:** 10.1002/smll.201400970

**Published:** 2014-06-11

**Authors:** Liwu Zhang, Chia-Yu Lin, Ventsislav K Valev, Erwin Reisner, Ullrich Steiner, Jeremy J Baumberg

**Affiliations:** Cavendish Laboratory, Department of Physics, University of CambridgeCambridge, CB3 0HE, UK E-mail: lz335@cam.ac.uk; jjb12@cam.ac.uk; Department of Chemistry, University of CambridgeCambridge, CB2 1EW, UK E-mail: reisner@ch.cam.ac.uk

**Keywords:** water splitting, BiVO_4_, photonic crystals, plasmonic

## Abstract

Photo-electrochemical water splitting is a very promising and environmentally friendly route for the conversion of solar energy into hydrogen. However, the solar-to-H_2_ conversion efficiency is still very low due to rapid bulk recombination of charge carriers. Here, a photonic nano-architecture is developed to improve charge carrier generation and separation by manipulating and confining light absorption in a visible-light-active photoanode constructed from BiVO_4_ photonic crystal and plasmonic nanostructures. Synergistic effects of photonic crystal stop bands and plasmonic absorption are observed to operate in this photonic nanostructure. Within the scaffold of an inverse opal photonic crystal, the surface plasmon resonance is significantly enhanced by the photonic Bragg resonance. Nanophotonic photoanodes show AM 1.5 photocurrent densities of 3.1 ± 0.1 mA cm^−2^ at 1.23 V versus RHE, which is among the highest for oxide-based photoanodes and over 4 times higher than the unstructured planar photoanode.

## 1. Introduction

Developing artificial photosynthesis routes using solar energy to produce H_2_ or other fuels is an attractive scientific and technological goal to address the increasing global energy demand and to reduce the impact on climate change from energy production.[1–7] However, it is still a great challenge to develop efficient and robust semiconductor photoelectrodes for water splitting, because this involves satisfying multiple requirements. A promising metal oxide photo­electrode material for efficient water oxidation is bismuth vanadate (BiVO_4_), which has a direct bandgap of 2.4 eV and a suitable valence band position for O_2_ evolution.[8] BiVO_4_ was firstly reported by Kudo and coworkers as a photocatalyst for water oxidation.[9] Since then BiVO_4_ has been widely investigated as a visible-light-driven photocatalyst for water oxidation and organic compounds degradation.[10–14] BiVO_4_ has recently attracted extensive attention as a photoanode for photoelectrochemical water splitting.[15–21] Metal doping (W, Mo, etc.) has been reported to enhance the electronic conductivity of BiVO_4_ and thus prevent electrons from accumulating in the bulk of the electrode film, whereas surface modification with a cobalt-oxide based co-catalyst can suppress surface recombination by preventing holes accumulating close to the surface of the semiconductor.[16,18,22–26] Nevertheless, most of the photogenerated charge carriers recombine in the bulk of BiVO_4_ due to inefficient separation of the electron-hole pairs.[25] It is thus a great challenge to find efficient methods to suppress the bulk recombination to enhance the water splitting efficiencies with such photoelectrodes.

The main reason for the dominant electron-hole recombination in the bulk is the short diffusion length of photoexcited charge carriers. To address this problem, nanostructuring has been extensively studied, reducing bulk recombination by shortening the diffusion length for charge carriers.[17,27–31] However, nanostructuring can also increase surface recombination and lower the surface photovoltage.[32] Nanophotonic structures allow manipulating and confining light on the nanometer scale and provide new opportunities to improve the efficiency in photoelectrodes. Several nanophotonic structures are of particular interest for solar water splitting. An optical cavity can reduce the thickness of photoelectrodes without compromising their light absorption by trapping resonant light in ultrathin films.[32] Plasmonic metal nanostructures with surface plasmon resonances (SPR) can act as antennas to localize optical energy and control the location of charge carrier generation.[33–39] Photonic crystals show great potential in manipulating light based on photonic band structure concepts, in which near-bandgap resonant scattering and slow photon effects can enhance the interaction of light with a semiconductor.[40–43] Moreover, the synergistic combination of a photonic crystal with SPR by tuning the slow photon effect to overlap with the SPR, can maximize these effects, and may result in an efficient solar water splitting system. Recently, synergistic effects of photonic crystal and SPR have been observed in TiO_2_ photonic crystals infiltrated with Au nanoparticles, which show greatly enhanced activities for pollutant degradation and water oxidation due to increased light harvesting.[38,44–46] However, the coupling effect of slow photon in a photonic crystal to SPR has not yet been studied and the mechanism is thus unclear in a visible-light-active photoelectrode, where the situation is very different due to the overlap of photonic stop band and the light absorption band of the photoelectrode.

Here we have for the first time combined BiVO_4_ inverse opals with SPR effects to enhance the water splitting efficiency by improving and manipulating light absorption within the BiVO_4_ photoanode. By tuning the photonic structure, the coupling of BiVO_4_ photonic crystal and localized surface plasmon resonance of Au nanoparticles (NPs) is achieved. By adding an un-patterned semiconductor underlayer, the designed structure overcomes the critical issue of reflection losses of light. Within the scaffold of an inverse opal (io-) photonic crystal, the SPR effect is significantly enhanced by the photonic Bragg resonance. The performance of the photonic crystal in light absorption and charge carrier separation is further enhanced by the amplified localized surface plasmonic effect.

## 2. Results and Discussion

**Figure 1:**
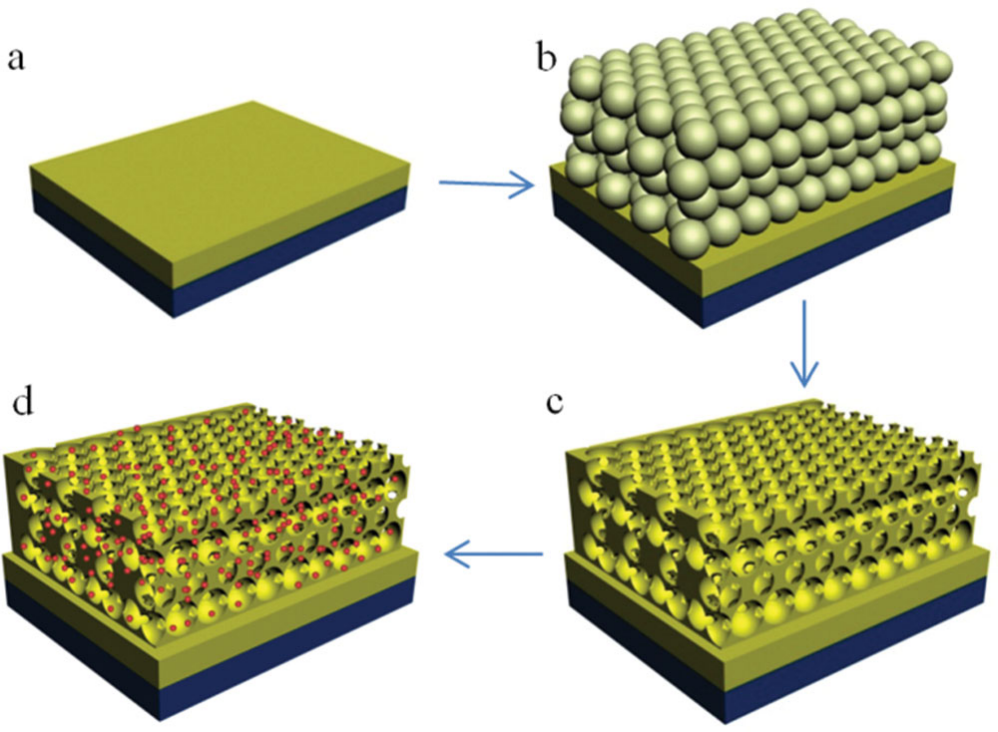
Illustration of synthetic strategy. a) An un-patterned layer of Mo:BiVO_4_ with thickness of 150 nm is deposited on FTO-coated glass. b) A colloidal crystal template is formed on the top surface by evaporation-induced self-assembly. c) The precursor is infiltrated, and the template removed to form an inverse opal structure. d) Surface plasmon effects are introduced by infiltrating Au NPs into the io-Mo:BiVO_4_.

The preparation of photonic nanostructured Mo:BiVO_4_ electrodes is illustrated in **Figure** **1**. First, an un-patterned 150 nm thick layer of Mo:BiVO_4_ was deposited onto fluoride-doped tin oxide (FTO)-covered glass by spin-coating. A colloidal crystal template of polystyrene beads was then formed on top of the planar Mo:BiVO_4_ surface by evaporation-induced self-assembly.[47] Subsequently, a homogenous amorphous complex precursor of BiVO_4_ was produced in aqueous solution by complexation of diethylenetriaminepentaacetic acid (DTPA) to low-cost Bi^3+^, V^5+^, and Mo^6+^. Mo (3 mol%) was added into the precursor to produce Mo-doped BiVO_4_ (Mo:BiVO_4_) for improved charge carrier transfer. Dip-coating was employed to infiltrate the Mo:BiVO_4_ precursor into the voids of the colloidal crystal template. After annealing in air at 500 °C for several hours, the template was removed while simultaneously crystallizing the io-Mo:BiVO_4_ framework. The photonic band gap of this io-Mo:BiVO_4_ photonic crystal can be easily tuned by changing the size of polystyrene spheres that form the colloidal template. To introduce plasmonic effects into the photonic crystal, gold nanoparticles were incorporated into the io-Mo:BiVO_4_ structure. A planar Mo:BiVO_4_ with similar thickness (≈1.5 μm) but without the inverse opal structure was also prepared for comparison under identical conditions.

**Figure 2:**
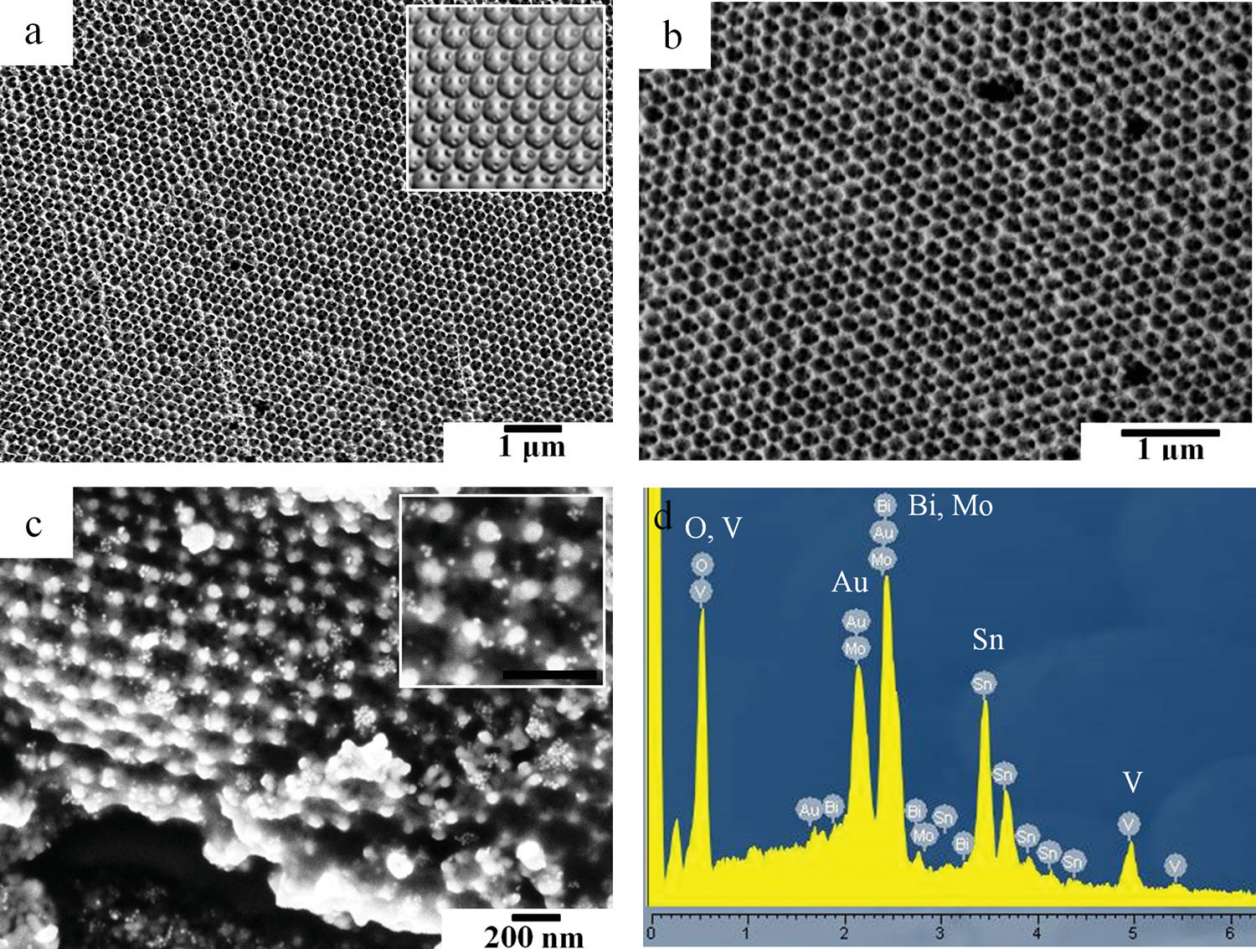
a,b) SEM images of io-Mo:BiVO_4_ prepared from colloidal crystal templates of polystyrene spheres with a) 260 nm diameter and b) 320 nm diameter. c) Cross-sectional SEM image of io-Mo:BiVO_4_ after introducing 20 nm Au NPs (The scale bar in the inset is 200 nm). d) EDS of the as-prepared io-Mo:BiVO_4_/AuNP (elemental Sn derives from FTO-coated glass). Inset of (a) is a computed top view inverse opal structure.

Typical scanning electron microscopy (SEM) images of the io-Mo:BiVO_4_ films produced from colloidal crystal templates of polystyrene spheres (PS) with 260 nm and 320 nm diameter are shown in **Figure** **2**. The well-ordered fcc inverse opal structure is clearly observed, demonstrating the successful infiltration of the Mo:BiVO_4_ precursor solution into the template and formation of the inverse opal structure with macropores of an average diameter of ≈200 nm, indicating ≈20% shrinkage during removal of the template by calcination. The size of macropore in the inverse opal can be easily tuned by using PS with different sizes to form the colloidal crystal template: an io-Mo:BiVO_4_ with pore size of 240 nm (Figure 2b) can be obtained by using PS with a diameter of 320 nm. In both Figure 2a and 2b, periodic order and full macropore interconnectivity is observed. This continuous porous structure enables subsequent introduction of Co–Pi and infiltration with Au NPs as well as allowing unhindered electrolyte diffusion throughout the entire photoanode. An SEM image of io-Mo:BiVO_4_ with lower magnification, showing larger areas of the inverse opal structure, is given in the Supporting Information (Figure S1). Figure 2c shows a cross-sectional SEM image of the inverse opal after loading with 20 nm Au NPs (of 2.2 × 10^−5^ g cm^−2^). The thickness of the inverse opal film is 1.5 μm. The Au NPs infiltrate deep into the inverse opal film, and are uniformly distributed and anchored on the Mo:BiVO_4_ surface. SEM images of BiVO_4_ planar films modified with Au NPs also show a uniform dispersion of Au NPs on the BiVO_4_ surface (Figure S2, Supporting Information). The elemental composition of the inverse opal film was analyzed by energy dispersive spectrometry (EDS) (Figure 2d), indicating the presence of Bi, V, Mo, O, and Au elements in the sample shown in Figure 2c.

**Figure 3:**
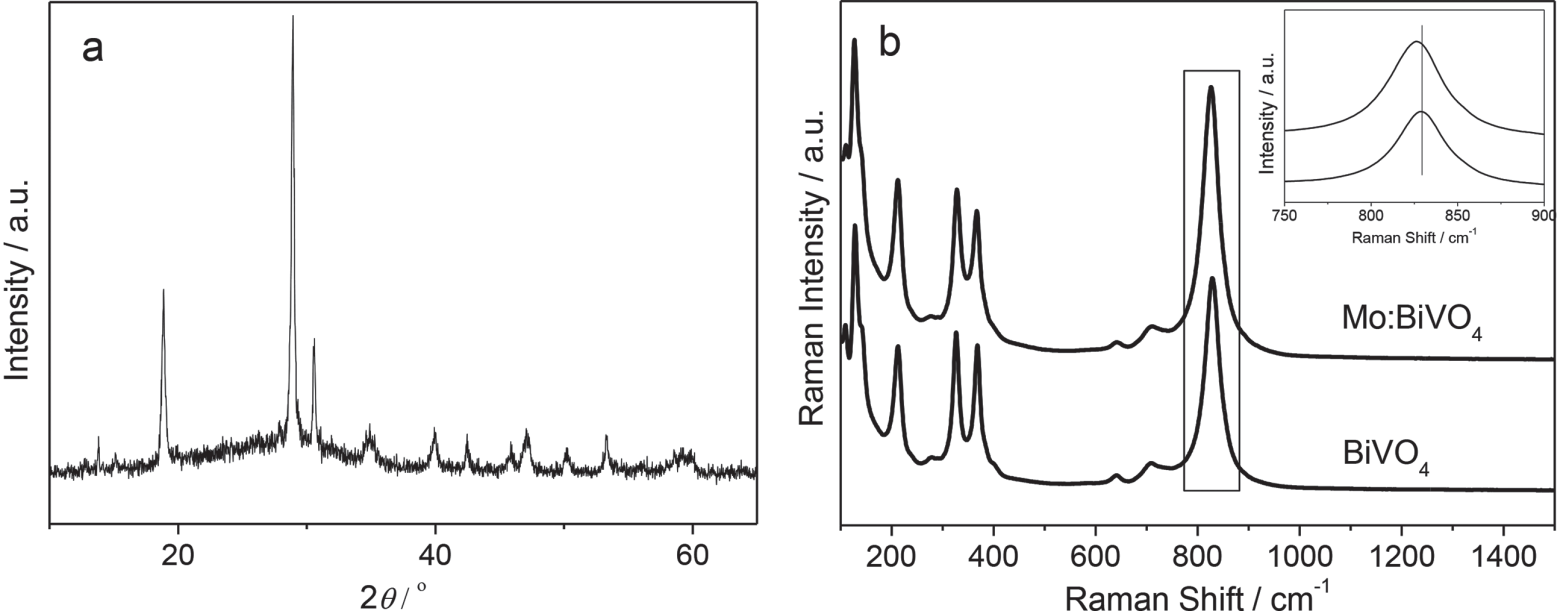
a) XRD pattern of the io-Mo:BiVO_4_, b) Raman spectra of the io-Mo:BiVO_4_, and c) Raman spectra of Mo:BiVO_4_ with and without Au NPs.

The X-ray diffraction (XRD) patterns of the io-Mo:BiVO_4_ films (**Figure** **3**a) can be matched to monoclinic BiVO_4_ (JCPDS No. 14–0688). The 2*θ* diffraction peaks of 28.8°, 30.5°, 35.2°, 39.8°, and 42.5° can be respectively indexed as (112), (004), (020), (211), and (015) planes of monoclinic BiVO_4_ structure, which is consistent with the literature.[16] No noticeable peaks from any secondary phases can be observed in the XRD pattern, indicating that Mo is substitutionally incorporated in BiVO_4_ facilitated by the very similar ionic radii of V^5+^ (0.36 Å) and Mo^6+^ (0.41 Å).[18] Raman spectra of these io-Mo:BiVO_4_ help identifying the doping sites in the crystal lattice (Figure 3b). The Raman mode at 829 cm^−1^ can be assigned to the symmetric stretching mode of VO_4_ units.[48] In io-Mo:BiVO_4_, the symmetric stretching mode shifts to lower wave number (826 cm^−1^) because Mo^6+^ (95.9 g mol^-1^) is heavier than V^5+^ (50.9 g mol^−1^), suggesting that Mo^6+^ substitutes V^5+^ in the VO_4_^3−^ tetrahedron.

**Figure 4:**
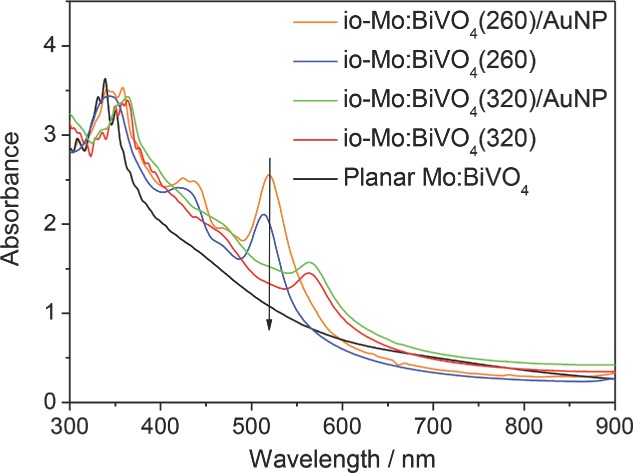
UV–Vis extinction spectra of inverse opals (io-) with/out Au NPs.

The optical absorption of the io-Mo:BiVO_4_ before and after further modification with 20 nm Au NPs was investigated (**Figure** **4**). In order to provide comparison with the water splitting experiments, these electrodes were immersed in water for 10 s before optical measurements. BiVO_4_ possesses an electronic band gap of 2.4 eV, corresponding to a wavelength of around 520 nm. The extinction from 520 nm to 900 nm of the Mo:BiVO_4_ films can be attributed to light scattering by the films. Compared to the planar Mo:BiVO_4_ film, the inverse opal samples exhibit additional peaks in the spectra. These extinction peaks are attributed to Bragg reflection at wavelengths matching the photonic stopband. For the io-Mo:BiVO_4_ (260 nm) film this Bragg peak is located at 513 nm, while the io-Mo:BiVO_4_(320) exhibits a red-shifted photonic stopband at 563 nm due to the larger pore size. The stopband follows a modified Bragg's law:[49]


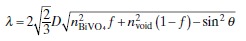


where *D* is the spherical pore diameter in the inverse opal, which is 200 nm for io-Mo:BiVO_4_ (260 nm), *n*_BiVO4_ and *n*_void_ represent the refractive indices of BiVO_4_ (*n*_BiVO4_ = 2.4) and void (*n*_void,water_ = 1.33) respectively, *f* is the volume fraction occupied by BiVO_4_ in the inverse opal (*f* = 0.2), and *θ* is the incident angle of light (at normal incidence in Figure 4). From the measured pore sizes in SEMs, the photonic stopbands of io-Mo:BiVO_4_(260) and io-Mo:BiVO_4_(320) films are calculated to be 510 nm and 580 nm respectively, close to the positions observed in the extinction spectra. The well-defined photonic stopbands in the io-Mo:BiVO_4_ indicate the highly ordered structure of the film, but the limited stopband width arises from residual imperfections. We note that the integrated extinction from 400–900 nm increases by only 11% from the planar film in the 260 nm inverse opal.

The extinction spectrum of io-Mo:BiVO_4_(260) after infiltrating with 20 nm Au NPs, designated as io-Mo:BiVO_4_(260)/AuNP, (Figure 4) shows the photonic stopband red-shifting by 6 nm to 519 nm, while the resonant extinction increases by 21% around 520 nm due to the localized surface plasmon resonance (LSPR) of Au NPs. In addition, the extinction is enhanced throughout the 450 to 600 nm range. In the middle of the stopband, the spatial distribution of the optical field becomes maximal at the surface of the BiVO_4_ micropores at precisely the location of the Au NPs, and the optical cross section of these plasmonic NPs is also maximized at this spectral position. This design thus optimizes optical fields at the photocatalytic surface. The extinction peak arising from Bragg reflection remains sharp and almost unchanged in shape, showing that the uniform coverage of Au NPs does not destroy the multiple photon interference that creates the stopband. A similar result is observed in the case of io-BiVO_4_(320) after Au NP infiltration, while a minimal red-shifting (2 nm) of photonic stopband is found.

**Figure 5:**
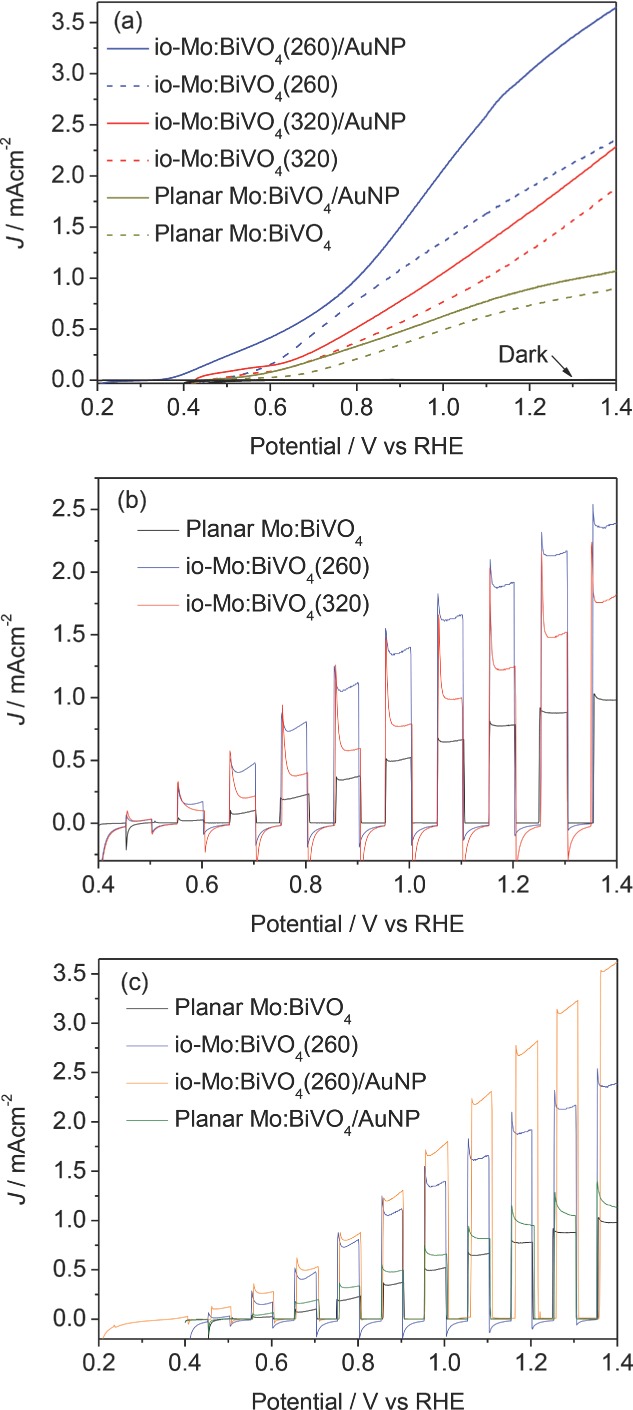
a) LSV under AM 1.5G illumination (100 mW cm^−2^) and dark response (dash lines). b) LSV recorded under chopped illumination. c) LSV of the electrodes with and without Au NPs recorded under chopped illumination. All runs were recorded with a scan rate of 10 mV s^−1^ at 25 °C in an aqueous phosphate buffer as electrolyte at pH 7.

The photoelectrochemical (PEC) response of the planar and io-Mo:BiVO_4_ electrodes was studied both in the dark and under AM 1.5G illumination (100 mW cm^−2^) in aqueous pH 7 phosphate buffer. A conventional three-electrode configuration was used with io-Mo:BiVO_4_ photoanode (working electrode), Pt wire (counter electrode), and Ag/AgCl_(KCl)_ reference electrode. All potentials are quoted verses the reverse hydrogen electrode (RHE). Linear sweep voltammetry (LSV) (recorded at a scan rate of 10 mV s^−1^) of the planar and io-Mo:BiVO_4_ photoanodes (**Figure** **5**a) shows photocurrents increase steadily with increasing applied positive potential under illumination, whereas the currents are negligible in the dark. The inconsistent shape of the photocurrent curves presumably results from the difference in resistance of the films. For the planar electrode, the photocurrent onset potential is found at 0.47 V, whereas the inverse opal electrodes show a decreased onset potential at 0.44 V. The inverse opal electrodes show a better performance than planar ones over the entire potential range from 0.44 to 1.4 V. At 0.6 V, the photocurrent densities of io-Mo:BiVO_4_(260) and io-Mo:BiVO_4_(320) are 160 ± 20 μA cm^−2^ and 90 ± 10 μA cm^−2^, which are 8 and 5 times, respectively, higher than the planar Mo:BiVO_4_ electrode (20 ± 10 μA cm^−2^). The photocurrent density of io-Mo:BiVO_4_(260) (2.0 ± 0.1 mA cm^−2^) is higher by more than a factor of two compared to the planar electrode (0.76 ± 0.06 mA cm^−2^) at 1.23 V. The io-Mo:BiVO_4_(260) shows a higher photocurrent density than the io-Mo:BiVO_4_(320) electrode over the entire potential range even though they have comparable integrated extinction, implying that the water oxidation performance is subtly dependent on the pore size inside the inverse opal. The LSVs of the photoanodes under chopped illumination were also studied and show a similar trend (Figure 5b).

The io- and planar Mo:BiVO_4_ electrodes were further surface-modified with 20 nm Au NPs (loading 2.2 × 10^−5^ g cm^−2^). The corresponding photocurrent responses (Figure 5a) show that the photocurrent onset potential is shifted negatively by more than 0.1 V after Au NPs infiltration, indicating more energetic plasmonic electrons injected from Au NPs (vide infra). For the io-Mo:BiVO_4_(260) electrode, the photocurrent density is increased from 2.0 ± 0.1 mA cm^−2^ to 3.1 ± 0.1 mA cm^−2^ at 1.23 V, thus enhanced by 55% (considerably more than the enhancement in extinction). This AuNP enhancement is not as strong in the case of io-Mo:BiVO_4_(320) and planar electrodes with only 26% and 21% increase at the same potential, respectively. The LSV under chopped illumination also confirms this observation (Figure 5c), indicating that the influence of Au NP plasmonic effects is intensified in the inverse opal structures. The photocurrent spikes are related to the generation and recombination dynamics of charge carriers in the photoanodes before reaching steady-state kinetics.

The io-Mo:BiVO_4_(260)/AuNP nanophotonic photoanode shows a photocurrent density of 3.1 ± 0.1 mA cm^−2^ at 1.23 V versus RHE, which is among the highest AM 1.5 (100 mW cm^−2^) photocurrents for photoanodes based on metal oxide.[16,22,50,51] The photocurrent we observe is four times higher than that of the corresponding planar Mo:BiVO_4_ photoanode (0.76 ± 0.06 mA cm^−2^ at 1.23 V versus RHE; see also Figures S3 and S4 in the Supporting Information). Since the measured specific surface area of io-Mo:BiVO_4_(260) (81 m^2^ g^−1^) is 31% higher compared to the planar photoanode (62 m^2^ g^−1^, with the large surface area due to the porous structure obtained after removing the organic component in the precursor), this significant enhancement is mainly attributed to a synergistic effect of the photonic inverse opal structure and plasmonic effects.

**Figure 6:**
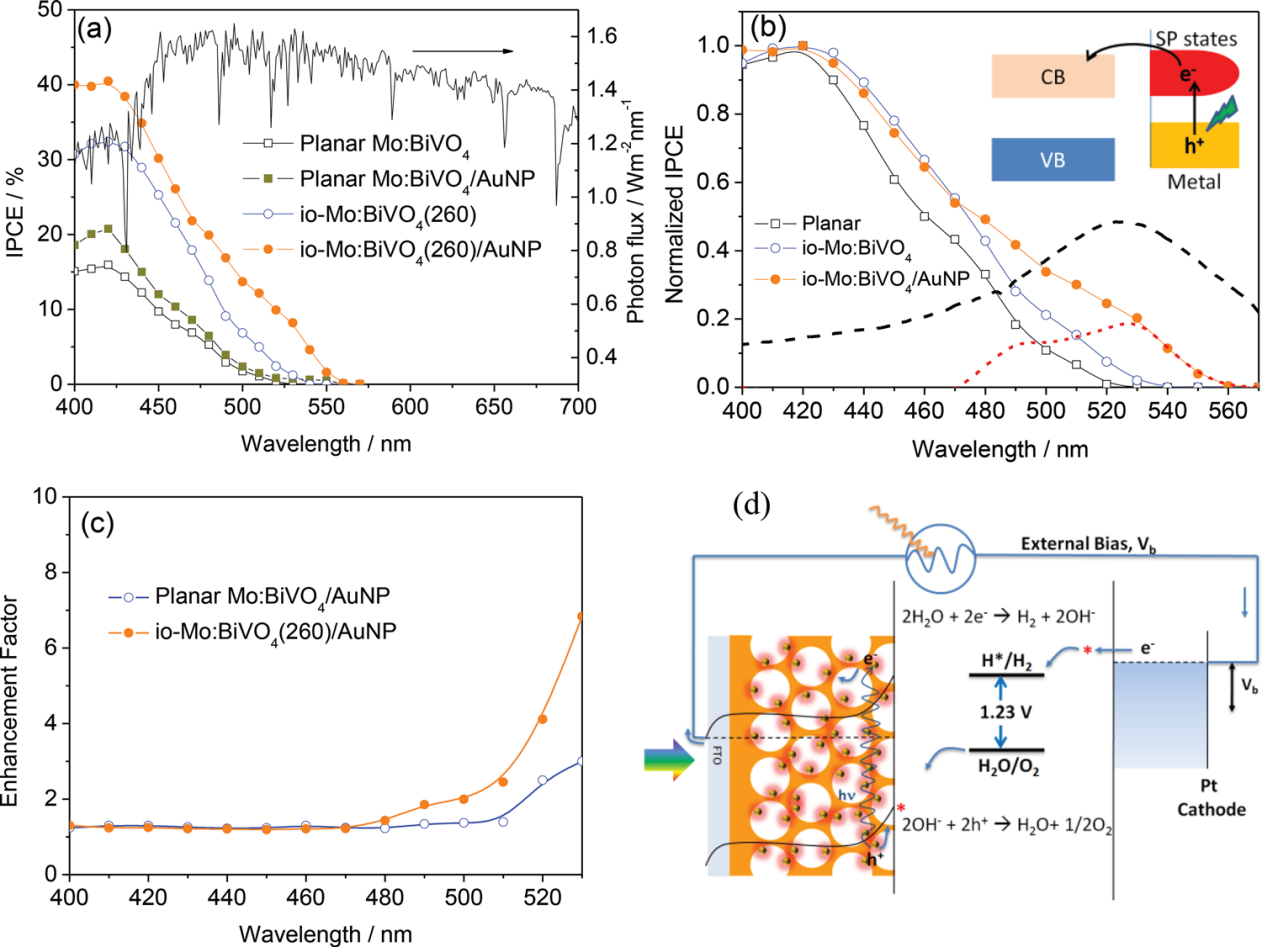
a) IPCE spectra of the different electrodes at an applied potential of 1.1 V and the AM 1.5 G 100 mW cm^−2^ solar spectrum. b) IPCE normalized to the IPCE maximum at 420 nm, red dash line is the difference in normalized IPCE spectra after Au NPs infiltration, black dash line shows localized surface plasmon extinction of the 20 nm Au NPs. Inset is an illustration of LSPR-induced charge transfer mechanism. c) IPCE enhancement factor (see text). d) Schematic illustration of solar water splitting with io-Mo:BiVO_4_/AuNP nanophotonic photoanode.

In order to clarify the effect of band-edge “slow photon” and plasmonic light scattering in the inverse opal before and after coating with Au NPs, incident photon-to-electron conversion efficiency (IPCE) measurements were performed (**Figure** **6**a). The IPCE spectra of the planar and inverse opal electrodes both exhibit a photoresponse up to 530 nm, as expected from the absorption edge of BiVO_4_. However after adding 20 nm Au NPs an additional strong photoresponse is seen from 530 to 560 nm caused by the localized surface plasmonic absorption. For all the electrodes, maximum IPCE is achieved at 420 nm, and found to be 16%, 33%, and 41% for the planar, inverse opal and inverse opal/AuNP electrodes, respectively.

The enhancements in IPCE are much stronger for *λ* > 490 nm, with 700% enhancement for io-Mo:BiVO_4_(260) at 520 nm. This coincides with the photonic stop band observed in extinction (Figure 4) in which the band gap multiple scattering and band-edge “slow light” effects can enhance the interaction of light with the semiconductor. The decreased onset potential of inverse opal electrode as compared with the planar electrode (Figure 5) can also be attributed to the enhanced light absorption, which increases the rate of e^−^/h^+^ formation and thus increases the photovoltage. The stop band position of the photonic crystal should be optimally near the absorption edge of BiVO_4_ (*λ* = 530 nm) to maximize the effect of the photonic band structure. Using 260 nm PS templates resulted in a photonic stop band of io-Mo:BiVO_4_(260) located at 513 nm, suggesting the photonic band structure influences the light absorption of BiVO_4_. In the case of io-Mo:BiVO_4_(320), near band gap resonant scattering and slow photon effect cannot enhance the light absorption of BiVO_4,_ because the stop band is at 563 nm, which is beyond the light absorption range of BiVO_4_. We also note that in our backside illumination geometry, light near the BiVO_4_ bandgap is Bragg-reflected from the 260 nm-pitch inverse opal above it and thus double passes the un-patterned 150 nm-thick layer of BiVO_4_ deposited on the FTO glass substrate. The existence of this underlayer can thus ameliorate the loss of light reflected out of the inverse opal structured photoelectrode due to the photonic stop band. The presence of the underlayer can also block FTO from direct contact with the electrolyte, which prevents back recombination of charge carriers.[52] The photocurrent of the inverse opal photoanode without this underlayer is over 30% lower (Figure S5, Supporting Information), indicating the importance of this compact underlayer. However, thicker un-patterned underlayers may also affect the charge transfer from the inverse opal structure to FTO substrate.

For a semiconductor solar water splitting system, it has been reported that the Au NP coating can have several effects:[33] i) Plasmon-induced charge transfer, ii) field-enhanced electron-hole production, iii) resonant photon scattering at Au NPs, and iv) the Au NPs can also act as charge carrier recombination centers due to the direct contact. Firstly, the resonant photon scattering effect can be ruled out, because Mie Theory calculations show that scattering from 20 nm sized Au NPs is very low (calculation results are shown in Figure S6, Supporting Information). Strong resonant photon scattering normally occurs for Au NPs with diameter larger than 50 nm.[53] The catalytic effect of the Au NPs can also be ruled out due to the negligible dark currents observed (Figure S7, Supporting Information). The additional strong photoresponse after adding Au NPs from 530 to 560 nm, which is beyond the absorption edge of BiVO_4_ (530 nm), confirms that plasmon-induced charge transfer occurs from Au NPs to BiVO_4_. This charge injection mechanism is analogous to dye sensitization on a semiconductor. Photoexcited plasmons promote single electrons to high energy in the Au NP. These electrons have energy higher than the conduction band of the semiconductor, facilitating a transfer of electrons from the surface plasmon states to the conduction band of BiVO_4_ (inset in Figure 6b). To clarify the influence of Au NPs, the IPCE spectra were normalized by the maximum value at 420 nm (Figure 6b). The difference in normalized IPCE spectra between io-Mo:BiVO_4_ and io-Mo:BiVO_4_/AuNP is evaluated and plotted as a red dashed line in Figure 6b, together with the measured localized surface plasmon extinction of the 20 nm Au NPs plotted as a black dashed line. The match in enhanced normalised IPCE from 480–550 nm with the plasmon resonance confirms the effect of plasmons interacting with the photonic Bragg resonance.

In the current work, it is found that the photocurrent enhancement effect of Au NPs is much stronger in the case of inverse opal samples than for planar samples. Enhancement factors of Au NPs in inverse opal and planar films were derived by dividing the IPCE values with those on the io-Mo:BiVO_4_(260) and planar electrodes, respectively. It is shown in Figure 6c that, in both cases, at 400 nm < *λ* < 470 nm the enhancements are more or less constant, at about 20% enhancement in the IPCE. However, for *λ* > 470 nm the enhancements are more significant due to the stronger plasmonic effect of the Au NPs at this region. In this region the enhancement factor in the io-Mo:BiVO_4_(260) sample is also much higher than that of the planar sample. Our SEM images (Figure 2c) show that the Au NPs infiltrate deep into the inverse opal film, and are uniformly distributed on the BiVO_4_ surface. Similarly, SEM images of BiVO_4_ planar films coated with Au NPs also show a uniform dispersion of Au NPs on the BiVO_4_ surface (Figure S2). The higher enhancement factor from Au NPs in the io-Mo:BiVO_4_(260) sample cannot just be due to the lower surface area of the planar film since (Figure 5a) the enhancement is low (26%) in the io-Mo:BiVO_4_/AuNP(320) and also a disordered Mo:BiVO_4_/AuNP inverse opal electrode (Figure S8, Supporting Information), even though the Au NPs infiltrate deep into these films. The higher enhancement factor in io-Mo:BiVO_4_(260) is thus attributed to the synergistic effect of photonic Bragg resonances and SPR in the inverse opal structure, which can amplify the plasmonic effect of Au NPs. In the case of io-Mo:BiVO_4_/AuNP(320), the synergistic effect is much weaker due to its photonic stopband located at 563 nm, which does not match the SPR of Au NPs. Figure 6d shows a schematic illustration of solar water splitting with this amplified plasmonic effect in an io-Mo:BiVO_4_ photoanode.

**Figure 7:**
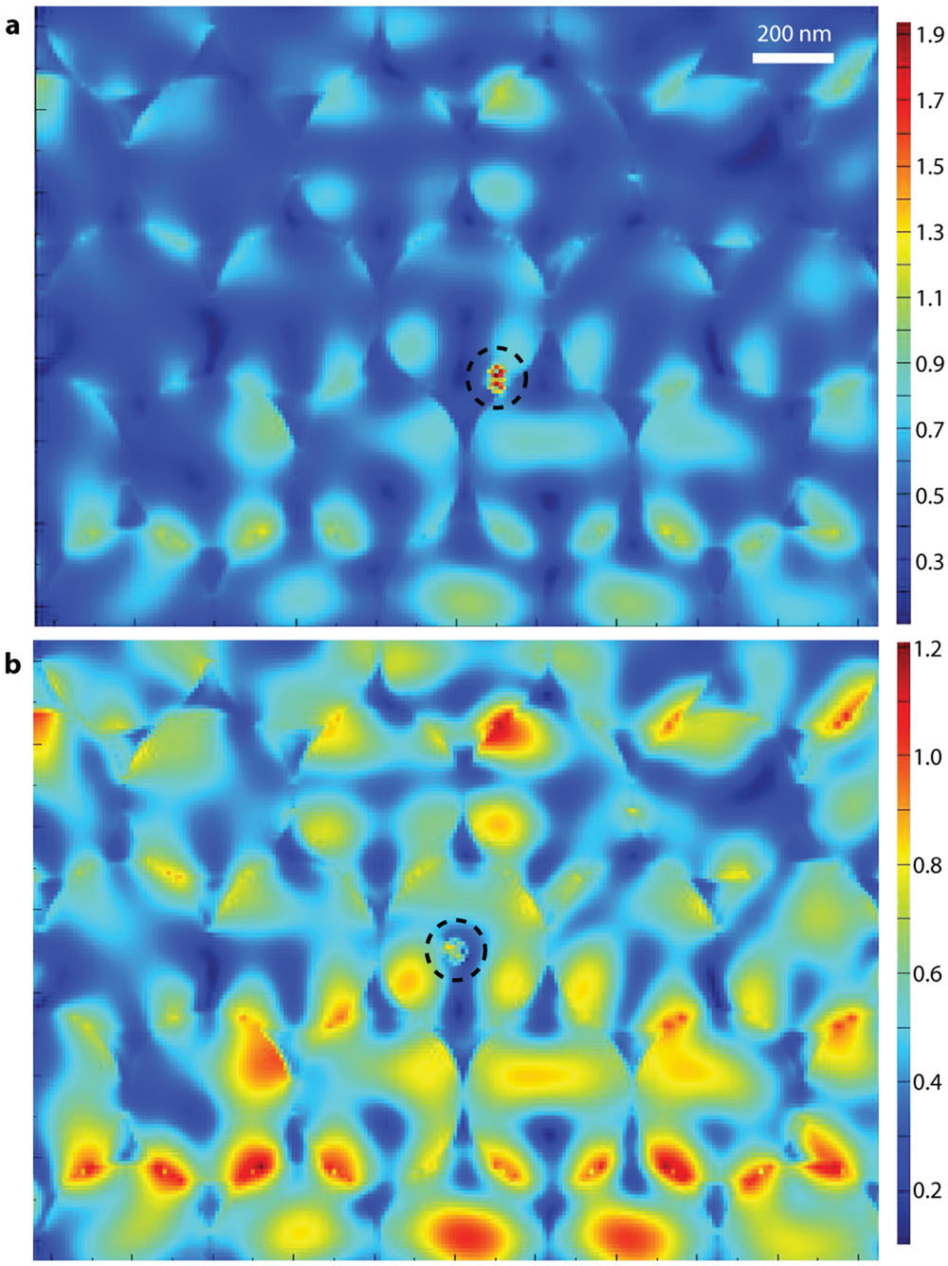
The simulated electric field distribution for gold nanoparticle (in the black dashed circles) modified BiVO_4_ inverse opal at *λ* = 533 nm. The 20 nm gold nanoparticle is either a) on the inverse opal wall, or b) in the centre of one of the pores in the inverse opal away from the wall.

To further explain this, we studied the interaction of plasmonic and photonic Bragg resonances in a gold nanoparticle modified io-BiVO_4_ by simulating with finite-difference time-domain (FDTD) methods. **Figure** **7**a shows the electric field distribution for the inverse opal with a 20 nm Au NP on the BiVO_4_ wall at *λ* = 533 nm. In the inverse opal structure, due to the photonic stop band, the incident light near the red edge of photonic band gap is localized near the high dielectric part (BiVO_4_) of the inverse opal, which strongly couples to the localized surface plasmonic resonance of the gold nanoparticles. In the simulation model of Figure 7b, the plasmonic resonance of the gold nanoparticle cannot couple with the photonic Bragg resonance as they are physically separated, consequently, the plasmonic resonance is much weaker than in Figure 7a. The coupling with the photonic Bragg resonance can significantly enhance the plasmon resonance by almost 4 times. This coupling effect is further confirmed by studying a disordered io-Mo:BiVO_4_/AuNP electrode, which shows a much lower photocurrent of 1.7 mA cm^−2^ at 1.23 V (Figure S8, Supporting Information). This significant decrease indicates the importance of Bragg photonic band structure in the current system, which provides a combination of “slow photon” effects, Bragg reflection, and more importantly, the coupling with the Au NP plasmonic resonance. It is notable that the loading of Au NPs is only 2.2 × 10^−5^ g cm^−2^ (area of the photoelectrode), which means only 0.22 g Au is needed for 1 m^2^ photoelectrode, indicating the amount of gold needed is actually very low.

**Figure 8:**
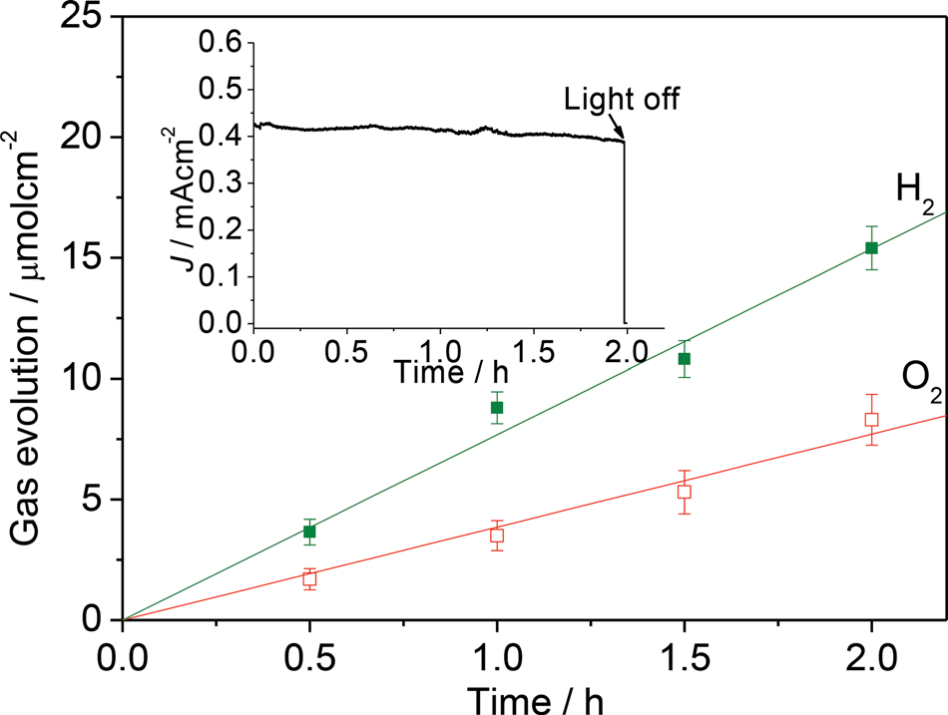
Gas evolution and the corresponding photocurrent transit (inset) of the io-Mo:BiVO_4_(260)/AuNP photoanode at an applied potential of 0.6 V (vs RHE).

To verify that the measured photocurrent of the nanophotonic photoanodes originates from water splitting rather than any other undesired side reactions, a water splitting experiment was performed at 0.6 V on the io-Mo:BiVO_4_(260)/AuNP photoanode, and the gas evolution and corresponding photocurrent response (**Figure** **8** inset) were measured (Figure 8). The ratio of evolution rates of H_2_ and O_2_ is close to the stoichiometric value of 2.0, with rates of 7.7 ± 0.2 μmol h^−1^ cm^−2^ for H_2_ and 3.8 ± 0.2 μmol h^−1^ cm^−2^ for O_2_. Assuming 100% Faradaic efficiency, at a photocurrent of 0.42 mA cm^−2^ the evolution rates of H_2_ and O_2_ should be 7.9 μmol h^−1^ cm^−2^ and 3.95 μmol h^−1^ cm^−2^, respectively. Hence the faradaic efficiencies for both gases are higher than 95%, indicating that the observed photocurrent can be fully attributed to water splitting. It is notable that the photocurrent only very slightly decreases by less than 5% after 2 h water splitting, implying the high stability of these nanophotonic photoanodes. The decay of photocurrent due to the trapping of O_2_ bubble in the electrode is not observed, which can be attributed to the continuous macroporous structure. The morphology of the recycled nanophotonic photoanode also shows no obvious change (Figure S9, Supporting Information).

## 3. Conclusions

In summary, we have prepared photonic nanostructured BiVO_4_ as highly-efficient photoanodes for solar water splitting. The superior performance obtained is attributed to a coupling of an inverse opal photonic crystal with localized surface plasmons from Au NPs that enhances the light absorption and charge carrier separation. The plasmonic effect of Au NPs is significantly amplified in the inverse opal structure due to a strong coupling with the photonic Bragg resonance. The reflection loss of light due to the photonic stop band in an inverse opal structure of visible-light-active semiconductor is avoided by simply adding an un-patterned semiconductor underlayer. The photonic nanostructured BiVO_4_ films results in AM1.5 photocurrents more than 4 times higher than equivalent planar electrodes. The nano-architecture of such photoelectrodes opens new opportunities to increase overall solar-to-H_2_ conversion efficiencies toward industrial viability by manipulating and confining light in the photoelectrode.

## 4. Experimental Section

*Synthesis of Mo:BiVO_4_ Precursor*: The amorphous complex precursor used for the infiltration of the void volume of the formed opal template is produced as follows: 0.02 mol of diethylenetriaminepentaacetic acid (DTPA) and 7.5 mL ammonia in water (13.0 mol L^−1^) were added to 200 mL hot distilled water. After dissolution, 10 mmol of Bi(NO_3_)_3_ (Sigma-Aldrich), 4.850 mmol of V_2_O_5_ powder (Sigma-Aldrich) and 0.043 mmol ammonium molybdate tetrahydrate (H_24_Mo_7_N_6_O_24_·4H_2_O, Sigma-Aldrich) were added. The resulting mixture was stirred and heated at 80 °C to promote the dissolution and reaction (complexation of Bi^3+^, V^5+^ and Mo^6+^ with DTPA) until the mixture turned into a transparent solution.

*Synthesis of the Opal Template*: First, an un-patterned layer of Mo:BiVO_4_ with thickness of 150 nm was deposited onto FTO glass by spin-coating. Monodisperse PS (polystyrene) spheres with diameters of 260 or 320 nm (Sigma-Aldrich) were diluted to 0.2 wt%. An FTO coated glass slide coated with 150 nm of Mo:BiVO_4_ was held vertically in a 10 mL vial containing the suspension of the monodisperse PS spheres. As the water evaporates and the meniscus sweeps down the substrate, capillary forces induce ordering of the spheres on the surface of the FTO glass slide.

*Synthesis of Inverse Opal (io-)Mo:BiVO_4_ Films*: To achieve a homogeneous infiltration of the transparent opal precursor, dip-coating infiltration was employed. The FTO-coated glass with 150 nm of Mo:BiVO_4_ un-patterned layer and opal template was dipped into the precursor, and this process repeated until the template was well infiltrated with the precursor. Finally, the templates and organic components in the precursor were removed by heating at 500 °C in air. A planar Mo:BiVO_4_ was prepared for comparison employing the same method but without opal template on the FTO glass slide.

*Infiltration of Au Nanoparticles into the io-Structure*: Commercially available Au NPs with size of 20 nm (EM.GC20, British Biocell International) were used to modify the io-electrode. Typically, 200 μL Au NP dispersion was drop cast onto the top surface of io-Mo:BiVO_4_, and the sample was kept in a 40 °C oven until dry. The planar Mo:BiVO_4_ films with Au NPs were prepared identically.

*Material Characterizations*: Scanning electron microscopy (SEM) images were taken using a LEO GEMINI 1530VP FEG-SEM. XRD data were recorded on a Phillips PW1800 diffractometer using a reflection geometry with variable divergence slits, CuKα_1,2_ radiation, and a secondary monochromator. Raman spectra were recorded on a Bruker Optic Senterra Raman Microscope-Spectrometer. UV-visible absorption spectra were measured on a HP 8453 UV-Visible Spectrophotometer. The UV–visible absorption spectra were measured after Co–Pi surface modification.

*Photoelectrochemical (PEC) Measurements*: Surface modification of the photoanodes with Co-oxide (Co–Pi) was performed by a photoassisted electrodeposition method.[25] Co–Pi was deposited at 0.4 V vs RHE for 5 min, with photocurrent densities of around 3 μA cm^−2^. PEC measurements were carried out with Co–Pi surface modified Mo:BiVO_4_ photoanodes on an electrochemical workstation (IVIUMSTAT potentiostat/galvanostat). A conventional three-electrode configuration was used with Mo:BiVO_4_, Pt wire and Ag/AgCl electrode as working, counter and reference electrodes, respectively. The electrolyte was an aqueous pH 7 phosphate solution (0.1 m). The working electrodes were illuminated from the back side. The light source was a 100 mW cm^−2^ solar light simulator (Newport Oriel, 150 W) equipped with an air mass 1.5 global filter and an IR water filter. For IPCE measurements an Oriel Cornerstone 130 monochromater was used. All the potentials are reported against the reversible hydrogen electrode (RHE) by using the equation *E*(V vs RHE) = *E*(V vs Ag/AgCl) + 0.059 pH + 0.197. The evolved gas was quantitatively analyzed using a gas chromatograph (Shimadzu 8A, TCD detector). The GC was equipped with a molecular sieve 5 A packed column.

*Simulations*: The numerical simulations were performed with Lumerical, a commercial FDTD Maxwell Equation solver. The simulated volume consisted of 9 inverse opal layers, whereby each layer comprised 9 rows of 9 inverted spheres. The ordering followed the fcc close-packing arrangement. Each sphere had a diameter of 200 nm and was filled with water. The gold nanoparticle was 20 nm in diameter. In both Figure 7a and 7b, the illumination was by plane polarized light. All the materials files used for the simulations were from Palik.

## Supporting Information

As a service to our authors and readers, this journal provides supporting information supplied by the authors. Such materials are peer reviewed and may be re-organized for online delivery, but are not copy-edited or typeset. Technical support issues arising from supporting information (other than missing files) should be addressed to the authors.
