# Supplementary material for: Plasmonic Enhancement in BiVO4 Photonic Crystals for Efficient Water Splitting
Source: Small. 2014 Jun 11;10(19):3970–8. doi: 10.1002/smll.201400970 (PMC4510818; doi:10.1002/smll.201400970)
Supplement: Supplementary file 1 — Supplementary [file smll0010-3970-sd1.pdf]

Copyright WILEY-VCH Verlag GmbH & Co. KGaA, 69469 Weinheim, Germany, 2014.

Supporting Information

for *Small.,* DOI: 10.1002/smll.201400970

Plasmonic Enhancement in $BiVO_4$ Photonic Crystals for Efficient Water Splitting

Liwu Zhang, Chia-Yu Lin, Ventsislav K. Valev, Erwin Reisner,* Ullrich Steiner, and Jeremy J. Baumberg**

Supporting Information

Significantly Enhanced Plasmonic Effect in $BiVO_4$ Photonic Crystals for Highly Efficient Water Splitting

Liwu Zhang[a], Chia-Yu Lin[b], Ventsislav K. Valev[a], Erwin Reisner[b], Ullrich Steiner[a], Jeremy Baumberg[a]

[a] Cavendish Laboratory, Department of Physics, University of Cambridge, CB3 0HE, Cambridge, UK.

[b] Christian Doppler Laboratory for Sustainable SynGas Chemistry, Department of Chemistry, University of Cambridge, CB3 0HE, Cambridge, UK.

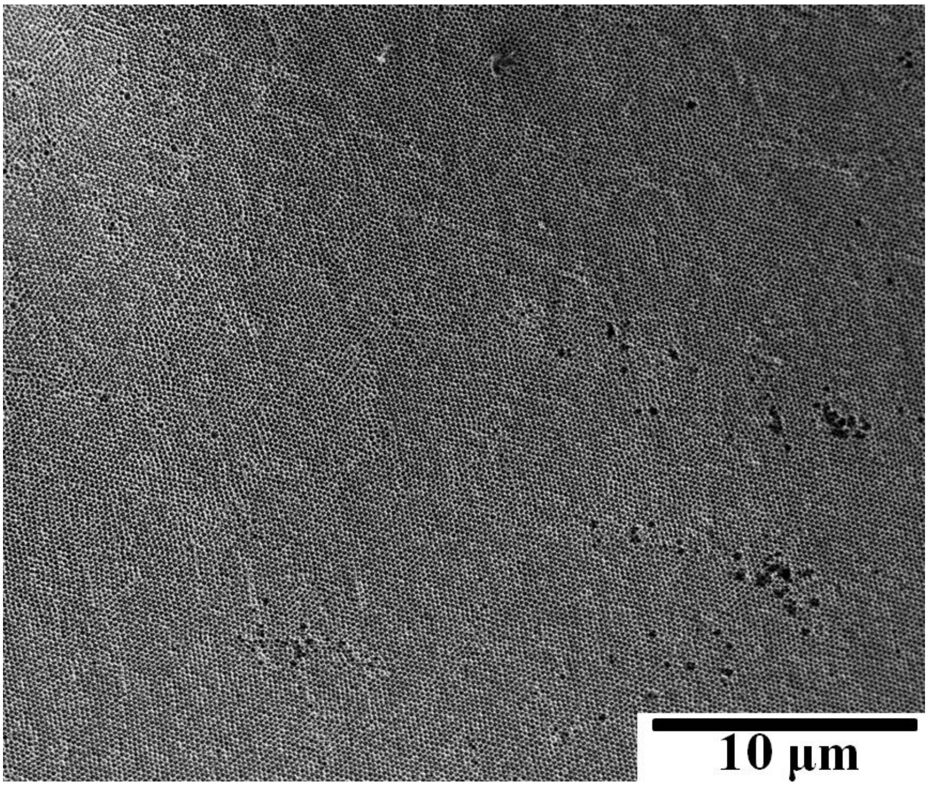

Figure S1. SEM image of io-Mo:$BiVO_4$(260) with lower magnification, showing larger area of the inverse opal structure.

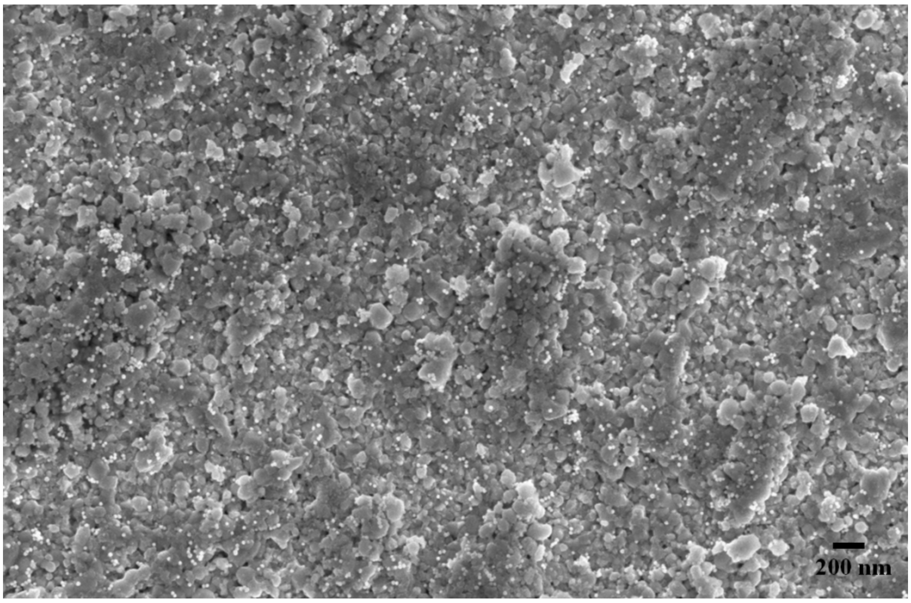

Figure S2. SEM image of Planar Mo:$BiVO_4$ modified with Au NPs.

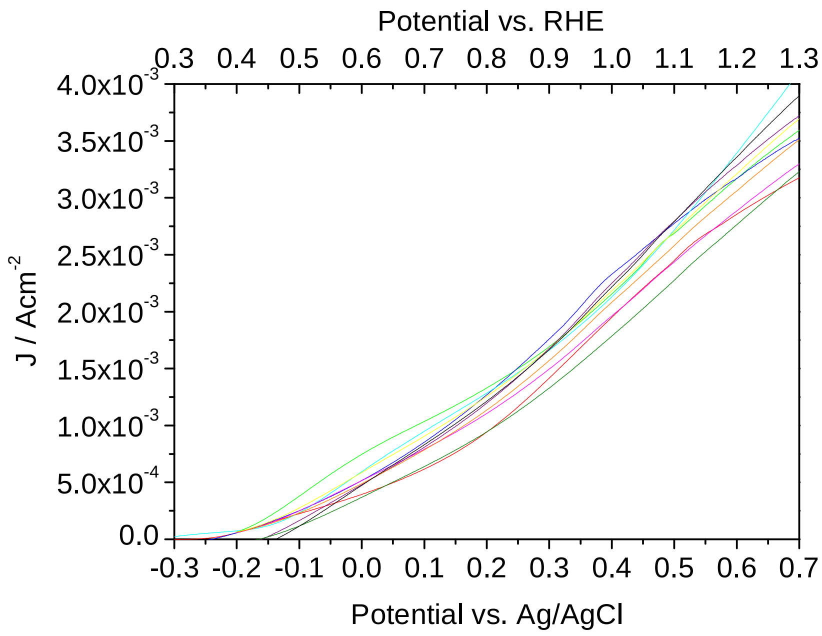

Figure S3. LSV recorded at 10 mV s^{-1} of the io-Mo:$BiVO_4$(260)/Au NP samples (10 samples have been overlaid together). Light source: AM 1.5G illumination, 100 mW cm^{-2}.

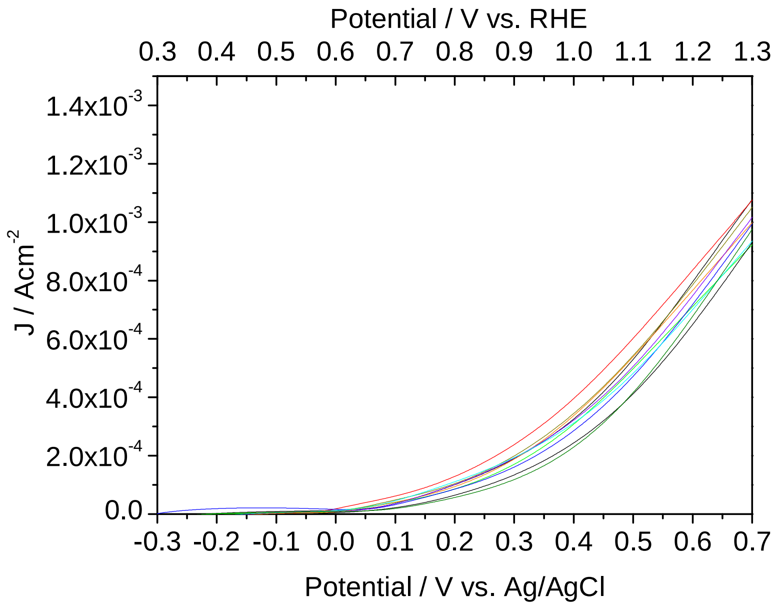

Figure S4. LSV recorded at 10 mV s^{-1} of unstructured planar Mo:$BiVO_4$ samples (10 samples have been overlaid together). Light source: AM 1.5G illumination, 100 mW cm^{-2}.

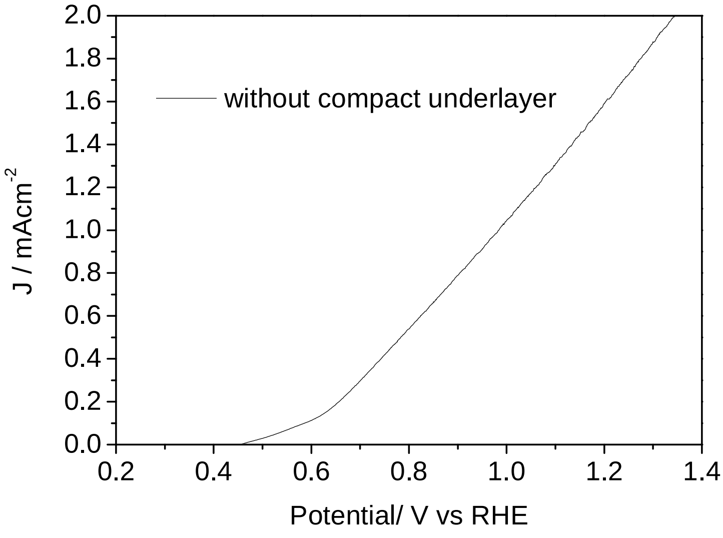

Figure S5. Linear sweep voltammetry recorded at 10 mV s^{-1} of the io-Mo:$BiVO_4$(260)/Au NP without 150 nm compact $BiVO_4$ underlayer in phosphate buffer (0.1 M, pH 7) in the dark and under illumination (100 mW cm^{-2}).

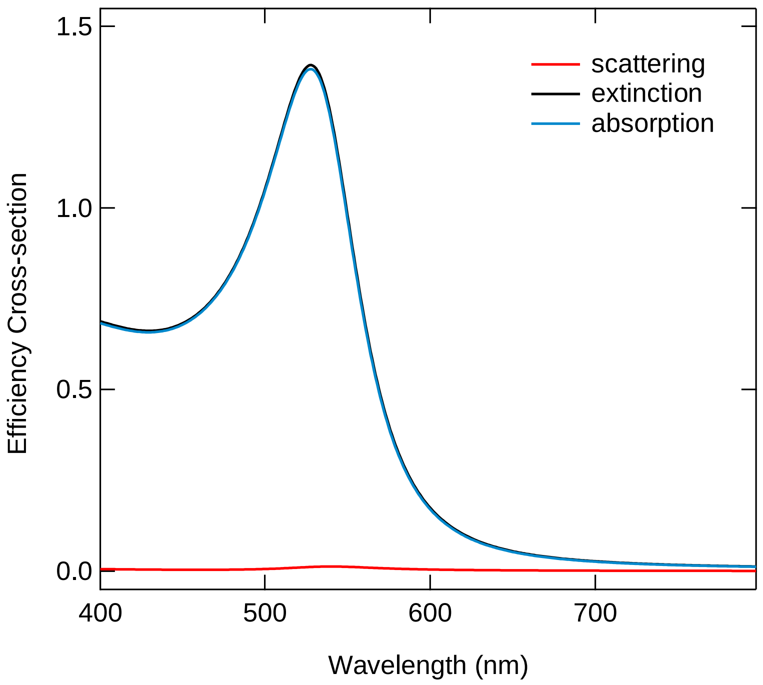

Figure S6. Mie Theory calculation showing the scattering, absorption and extinction per 20 nm Au NP vs. wavelength.

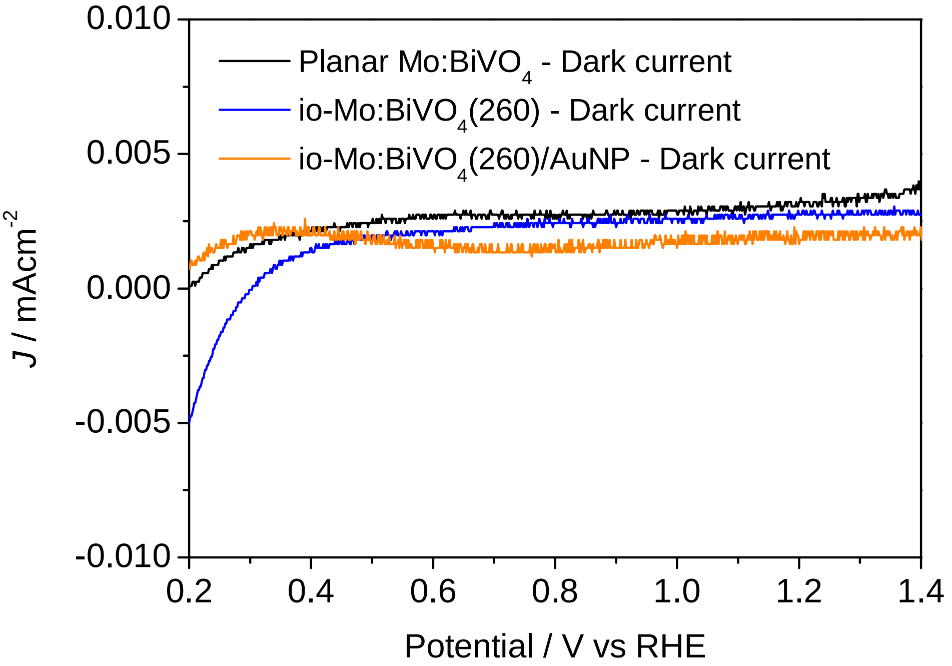

Figure S7. Linear sweep voltammetry of different electrodes recorded at a scan rate of 10 mV s^{-1} in phosphate buffer solution (0.1 M, pH 7) in the dark.

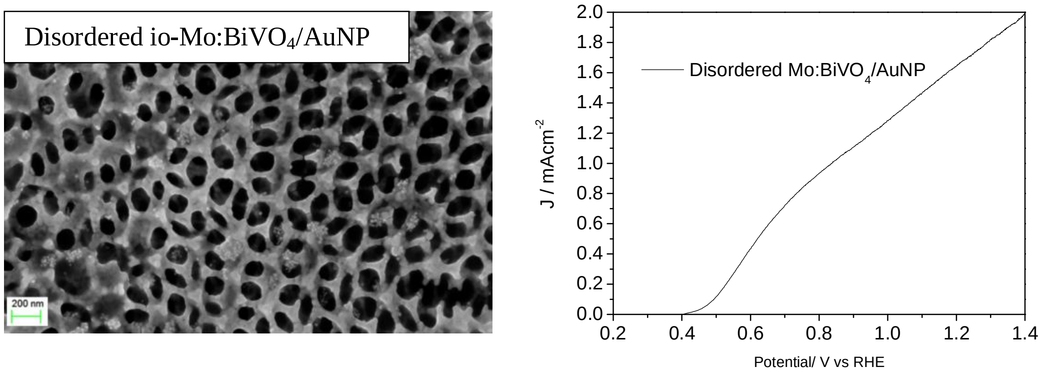

Figure S8. (a) SEM image and (b) the corresponding photocurrent of the disordered Mo:$BiVO_4$/Au NP inverse opal electrode, fabricated by using a template with mixed 260 nm and 100 nm sized polystyrene spheres. Photocurrent was recorded at a scan rate of 10 mV s^{-1} at 25 ^{o}C in phosphate buffer (0.1 M, pH 7) solution.

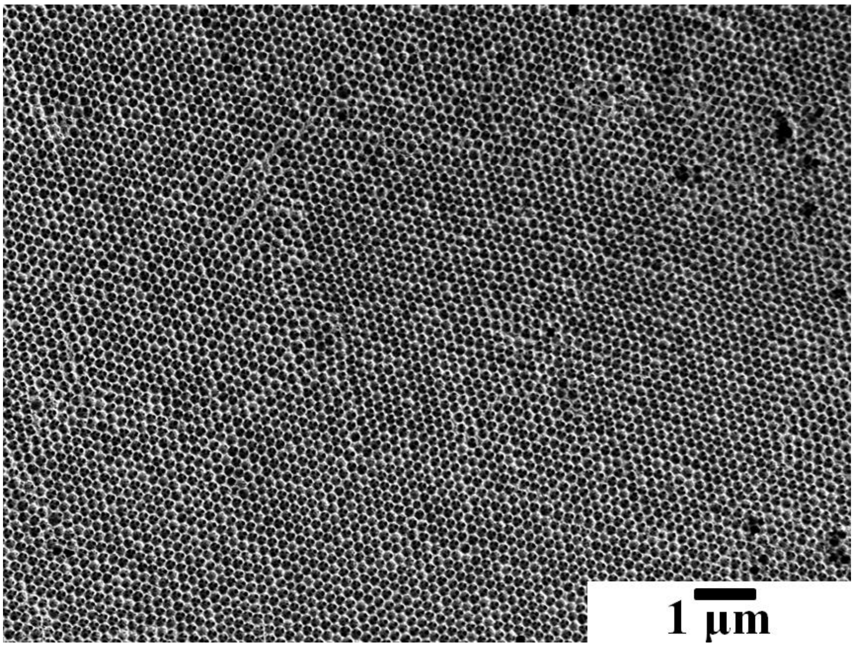

Figure S9. SEM image of the recycled nanophotonic photoanode after 2 hours water splitting.
